# A comprehensive review on the association and prevention of long-term COVID-induced heart failure: A review

**DOI:** 10.1097/MD.0000000000038736

**Published:** 2024-07-05

**Authors:** Zhangqing Ren, Gang Li

**Affiliations:** a Department of General Practice, Shaoxing People’s Hospital, Shaoxing, China.

**Keywords:** cardiovascular complications, heart failure, long-term COVID-19, myocardial injury, preventive strategies

## Abstract

The coronavirus disease 2019 (COVID-19) disease caused by the severe acute respiratory syndrome coronavirus 2 has had a widespread global impact. In addition to the main respiratory symptoms, research has found significant effects of this virus on the cardiovascular system. This article comprehensively explores the phenomenon of “long-term COVID-19” or postacute sequelae of severe acute respiratory syndrome coronavirus 2 infection, wherein some recovered patients continue to experience long-term health issues after the resolution of acute illness. We delve into the potential reasons behind these symptoms, including increased risk of heart disease, myocardial injury, abnormal inflammatory responses, thrombosis formation, and immune system dysfunction, among others. Furthermore, this paper highlights the potential association between long-term COVID-19 and HF (heart failure), and proposes corresponding preventive strategies. To address this, we advocate for a collaborative approach involving interdisciplinary teams for treatment and management.

## 1. Introduction

Long-term coronavirus disease 2019 (COVID-19), also known as postacute sequelae of severe acute respiratory syndrome coronavirus 2 infection (PASC), refers to the condition where patients experience discomfort for more than 3 months following infection with severe acute respiratory syndrome coronavirus 2 (SARS-CoV-2), with at least 2 months of symptoms that cannot be explained by other illnesses. According to the World Health Organization’s definition, long-term COVID-19 comprises 2 stages: the persistent symptomatic stage (4–12 weeks) and the post-COVID-19 syndrome stage (>12 weeks).^[1]^ These symptoms may persist for weeks or even months, significantly impacting the patient’s quality of life. Notably, some of these symptoms are related to the cardiovascular system, particularly the occurrence and exacerbation of HF.

The symptoms of long-term COVID-19 are diverse and can include persistent fatigue, reduced physical stamina, difficulty breathing, palpitations, chest pain, abnormal heart rate, low blood pressure, cognitive impairment, headaches, insomnia, muscle and joint pain, altered sense of smell and taste, as well as gastrointestinal symptoms such as nausea, vomiting, and abdominal pain. Additionally, abnormal symptoms may also manifest in the kidneys, liver, skin, and immune system.

Although the exact pathogenesis of long-term COVID-19 is not fully understood, preliminary research suggests that it may be associated with factors such as viral persistence, ongoing inflammatory response, immune system dysregulation, and vascular damage. Given the complexity of long-term COVID-19 symptoms, treatment strategies often require individualization and the involvement of a multidisciplinary team, including internal medicine, pulmonology, cardiology, rehabilitation, and mental health experts.

This review article meticulously investigates the intricate relationship between long-term COVID-19 and the onset of HF, with a keen focus on delineating the multifaceted cardiovascular complications attributed to PASC. It aims to: first, elucidate the complex pathophysiological mechanisms, including myocardial injury, heightened inflammatory responses, and the propensity for thrombosis formation; and second, propose a strategic framework for the prevention and management of these long-term cardiovascular sequelae. Through a rigorous synthesis of existing research, this piece endeavors to advocate for an integrative, multidisciplinary approach, highlighting the imperative for collaborative efforts in mitigating the cardiovascular aftermath of COVID-19.

## 2. Association analysis of long-term COVID-19 and HF

During the COVID-19 pandemic, several case reports and studies have indicated a certain association between long-term COVID-19 and HF. We conducted comprehensive searches on PubMed, Embase, and Google Scholar to retrieve relevant studies for our review. The following keywords and combinations were used: “COVID-19,” “long-term effects,” “heart failure,” “COVID-induced heart failure,” “SARS-CoV-2,” “cardiovascular complications,” “chronic heart failure,” “post-acute sequelae of SARS-CoV-2 infection (PASC),” and “prevention of heart failure.”

### 2.1. Overview of HF

HF, also known as congestive HF, is a clinical syndrome characterized by structural and/or functional abnormalities in the heart leading to impaired ventricular filling and/or ejection of blood. Its main pathophysiological features include pulmonary congestion and/or systemic congestion, with or without tissue organ hypoperfusion. The primary clinical manifestations of HF include dyspnea, fatigue (limited exercise tolerance), and/or fluid retention (peripheral edema), along with elevated plasma levels of natriuretic peptides. HF represents the final stage of most cardiovascular diseases and is characterized by high incidence, mortality, and rehospitalization rates.^[2–4]^

Significant progress has been made in the treatment of HF in recent years. In terms of pharmacotherapy, some newer drugs have shown favorable effects in HF with reduced ejection fraction (HFrEF), particularly across the full range of ejection fraction. Treatment paradigms have also shifted from the traditional “golden triangle” (beta-blockers, renin-angiotensin system inhibitors, and aldosterone receptor antagonists) toward newer therapeutic approaches. However, there is a lack of development of drugs targeting HF with preserved ejection fraction (HFpEF), and many have failed in clinical trials.^[5–7]^

Furthermore, there have been new concepts and advancements in the prevention and management of HF. For example, there are new consensuses on the definition, diagnosis, assessment, treatment, prevention, and management of worsening chronic HF, providing guidance for clinicians to identify and treat patients with worsening chronic HF early on.^[8]^

### 2.2. Increased risk of heart disease

Severe COVID-19 patients may experience transient pulmonary hypertension due to hypoxia, pulmonary vascular spasm, inflammation, and hypercapnia, leading to severe hypoxia and right ventricular ejection impairment. Additionally, myocardial ischemia, hypoxia, and direct myocardial injury can contribute to left ventricular dysfunction, exacerbating pulmonary congestion. COVID-19 infection can worsen existing heart conditions and lead to complications. The virus can affect the cardiovascular system through various mechanisms, including myocarditis, thrombosis formation, and cardiovascular damage. These factors increase the risk of HF. According to previous research and clinical reports, common cardiovascular complications in COVID-19 patients include acute myocardial injury (in 21% of patients), arrhythmias (in 10.4% of patients), and HF (in 2.8% of patients).^[9–12]^

The long-term sequelae of COVID-19 in recovered individuals remain unclear and may be overlooked. There are currently limited follow-up reports on postdischarge cardiovascular assessments. In a prospective study of 230 COVID-19 recovered patients previously hospitalized, researchers conducted a 2-month follow-up. Among these patients, 36 (16%) showed persistent or delayed onset of cardiovascular disease during the follow-up visit at 2 months. Of these, 62% of the recovered patients had already experienced cardiovascular disease during their hospital stay. Delayed cardiovascular complications included myocarditis, pericarditis, and ventricular dysfunction, which may lead to HF. In a 1-year telephone follow-up, 105 (45%) of the recovered patients reported ongoing symptoms, with the most common being dyspnea and fatigue, both of which are associated with HF. Sixty percent of the recovered patients exhibited persistent chest CT abnormalities, with 28% complaining of persistent cardiopulmonary symptoms during long-term follow-up.^[13]^ Besides, survivors of COVID-19 exhibit a significantly elevated 12-month incidence of cardiovascular diseases compared to controls without a history of the virus.^[14]^ Research indicates that the risk and 1-year burden of cardiovascular disease are substantial among survivors of acute COVID-19.^[15]^ Consequently, both clinicians and individuals with a prior COVID-19 infection must remain vigilant regarding long-term cardiovascular health monitoring and management.

Furthermore, the cardiovascular manifestations of long-term COVID-19 have garnered widespread attention. In addition to common symptoms like chronic fatigue, patients may also experience chest pain and abnormal electrocardiograms. Imaging studies of the heart and blood vessels have provided evidence of chronic and postinfection pericarditis, as well as subsequent left or right ventricular dysfunction. These research findings further support the potential risk of HF after SARS-CoV-2 infection.^[16]^

### 2.3. Myocardial injury

The European Society of Cardiology updated its acute and chronic HF diagnosis and treatment guidelines in 2023, building upon the 2021 guidelines with a focus on recent randomized controlled trials and meta-analyses in HF treatment. The updates primarily focus on the management and prevention of chronic HF (including HF with mildly reduced ejection fraction [HFmrEF] and HFpEF), acute HF, and complications of HF.

Regarding chronic HF, the 2023 European Society of Cardiology updated guidelines recommend the use of sodium-glucose cotransporter 2 inhibitors (such as dapagliflozin or empagliflozin) for symptomatic HFmrEF and HFpEF patients to reduce the risk of HF hospitalization or cardiovascular death. This recommendation is based on multiple studies, including EMPEROR-Preserved and DELIVER trials.

In the context of acute HF, the updated guidelines emphasize the importance of intensified treatment strategies in the early stages, both before discharge and postdischarge, for acute HF patients. This includes frequent and careful follow-up, initiation, and intensification of evidence-based therapies to reduce readmission and mortality rates.

Additionally, for HF patients with comorbid chronic kidney disease and type 2 diabetes, the new guidelines provide 2 new recommendations: first, the use of sodium-glucose cotransporter 2 inhibitors to reduce the risk of HF hospitalization or cardiovascular death, and second, the use of mineralocorticoid receptor antagonists in type 2 diabetes and chronic kidney disease patients to lower the risk of HF hospitalization.

The American Heart Association, American College of Cardiology, and HF Society of America released new HF management guidelines in 2022. These guidelines emphasize a patient-centered approach and provide essential references for clinicians from HF prevention to diagnosis, treatment, and management. The new guidelines elaborate on HF definition and classification, staging, and prevention, introducing new classifications such as HFmrEF and HFimpEF based on left ventricular ejection fraction.

Many COVID-19 patients exhibit elevated levels of high-sensitivity cardiac troponin I and creatine kinase-myocardial band, indicating myocardial injury.^[17]^ Myocarditis and direct damage to myocardial cells may be one of the reasons leading to HF, as COVID-19 can directly infect cardiac cells, causing myocardial injury. This direct damage is likely due to the interaction between the coronavirus and the angiotensin-converting enzyme 2 receptors on the surface of cardiac cells. The SARS-CoV-2 virus can directly infect cardiac cells, leading to viral myocarditis, which causes abnormal cardiac function and further contributes to viral myocarditis. Severe COVID-19 infection can result in cardiac and pulmonary dysfunction, with hypoxemia potentially increasing the burden on the heart, thereby triggering or worsening HF. Long-term COVID-19 may lead to myocardial injury, affecting patients’ prognosis.

In the study by Wei et al,^[18]^ they found that among 101 COVID-19 patients, 15.8% exhibited acute myocardial injury. These patients with acute myocardial injury were more likely to require admission to the intensive care unit and had a higher need for mechanical ventilation and vasopressor therapy. All deaths occurred in patients with acute myocardial injury. These research findings further support the possibility of myocardial injury resulting from long-term COVID-19, ultimately leading to HF.^[18]^

### 2.4. Abnormal inflammatory response

COVID-19 infection can trigger a systemic inflammatory response, which may have negative effects on the cardiovascular system, especially in severe cases. The intense inflammatory response induced by COVID-19 infection in critically ill patients can lead to cell damage and the release of inflammatory mediators, potentially impairing cardiac function and causing HF.

Long-term COVID-19 can lead to a cytokine storm, characterized by increased levels of proinflammatory cytokines such as interleukin (IL)-1β, IL-6, IL-12, interferon-inducible protein 10, and monocyte chemoattractant protein-1 in critically ill patients with COVID-19, resembling a cytokine storm-like profile.^[19,20]^ The inflammatory response and cytokine release induced by COVID-19 may adversely affect cardiac function, as excessive inflammation and cytokine release can lead to vascular endothelial dysfunction, myocarditis, and cardiac dysfunction. COVID-19 infection triggering a systemic inflammatory response may result in inflammation and damage to the cardiovascular system, ultimately leading to the development of HF.

### 2.5. Thrombosis

After COVID-19 infection, direct viral damage to vascular endothelial cells and the indirect effects of inflammation leads to endothelial dysfunction. Endothelial cells play a crucial role in regulating vascular tone, maintaining blood flow, and inhibiting thrombus formation. Endothelial dysfunction may result in the formation of microangiopathy, a disorder of microcirculation is characterized by microvascular spasm and/or obstruction, leading to tissue hypoxia and organ damage.

Furthermore, endothelial injury and inflammation promote the activation of the coagulation system, increasing the risk of thrombosis. Elevated levels of procoagulant factors and fibrin degradation products in the blood of COVID-19 patients indicate the presence of a prothrombotic state. These changes may lead to the formation of in situ thrombi, particularly in the microvasculature, due to endothelial damage and alterations in blood flow dynamics.^[21]^ Moreover, COVID-19 infection can precipitate thrombosis in atypical vascular sites, further complicating the clinical picture. Extensive vascular thrombosis, notably within the portal and sinusoidal vessels, has been observed, suggesting a broader endothelial involvement across different vascular beds.^[22]^ Additionally, superficial vein thrombosis, while less common, has also been reported in COVID-19 patients, indicating the extensive nature of coagulopathy.^[23]^ Pulmonary embolism, a critical and potentially fatal complication, emerges as another manifestation of thrombosis in COVID-19, reflecting the severe impairment in both the macrovascular and microvascular systems.^[24]^ These observations underscore the necessity of vigilant monitoring and aggressive management of thrombotic complications in patients with COVID-19, especially those presenting with atypical thrombotic events.

COVID-19 infection is associated with an increased risk of thrombosis, and blood clots can lead to cardiovascular events, including myocardial infarction and HF. COVID-19 patients may experience a hypercoagulable state, which can increase the risk of thrombosis.^[25]^ Cardiac thrombosis can result in myocardial infarction, angina, and HF, posing a high risk of thrombosis formation. Thrombosis within the cardiac vasculature can cause coronary artery disease, leading to inadequate myocardial blood supply and HF. This is particularly dangerous for COVID-19 infections in patients with preexisting HF, who often have underlying cardiovascular disease and suffer additional cardiac stress and damage due to the infection.^[26]^

Microclots detected in long-term COVID cases also contribute to thrombosis formation. COVID-19 infection may induce thrombosis, further leading to HF. Clots can obstruct microvasculature, hinder oxygen exchange, and cause myocardial hypoxia and damage. In the study by Pretorius et al,^[27]^ they found a significant presence of microclots in plasma samples from patients with long-term COVID-19/PASC, showing resistance to fibrinolysis. They also observed elevated levels of various inflammatory molecules, including α(2)-antiplasmin, various fibrinogen chains, and serum amyloid A in plasma samples during the acute phase of COVID-19 infection and in the long-term post phase. These research findings suggest that COVID-19 infection may trigger an antifibrinolytic response, leading to the formation of resistant blood clots, further influencing the development of HF.^[27]^

### 2.6. Immune system abnormalities

COVID-19 can trigger immune system abnormalities, and in severe cases, there may be an explosive inflammatory response, potentially leading to multiorgan dysfunction. Research has found that a significant portion of COVID-19 patients develop autoantibodies targeting the heart. Fagyas et al^[28]^ reported in their study that 68% of the 104 severe COVID-19 patients tested positive for cardiac autoantibodies. These autoantibodies might contribute to an immune response that delays the recovery process from COVID-19 and provides a new research perspective on long-term COVID-19 complications. These findings suggest that SARS-CoV-2 infection can trigger immune-mediated cardiac injury or autoimmune myocarditis, ultimately leading to HF.^[28]^

### 2.7. Vascular injury

COVID-19 may cause vascular injury through various mechanisms. One major possible mechanism is the entry of the novel coronavirus into host cells via the ACE2 receptor, which is highly expressed in endothelial cells. Once the virus invades, it can directly damage endothelial cells, leading to dysfunction and structural disruption of blood vessels. Additionally, COVID-19 infection may trigger an overreactive immune response, causing systemic inflammation, which can further contribute to vascular injury. Vascular damage can result in inadequate blood supply and increased workload on the heart, leading to HF. Furthermore, a vascular injury may induce inflammation and blood clot formation, further obstructing blood flow and burdening the heart, ultimately leading to HF. Endothelial cell damage can lead to microcirculation abnormalities, increased heart weight, and endocardial thickening, all of which may contribute to HF.^[29]^

Hachim et al^[30]^ research, analyzing publicly available datasets, revealed the molecular mechanisms through which COVID-19 infection may cause vascular injury. They found that certain genes associated with endothelial cells and vascular biology were significantly upregulated in lung tissues infected with the novel coronavirus, suggesting an important mechanism for COVID-19-induced vascular injury.^[30]^

Furthermore, modulating the renin-angiotensin system may offer an effective approach to ameliorating HF in COVID-19 patients. Activating the ACE2/Ang-(1-7)/Mas receptor pathway or inhibiting the ACE/Ang II/AT1R axis has shown promise in therapeutic strategies aimed at reducing the progression of HF. By utilizing ACE inhibitors to decrease the formation of angiotensin II, the subsequent interactions with the AT1 receptor are minimized, thereby reducing its deleterious effects on cardiac function.^[31]^ This approach not only helps in mitigating the direct vascular damage caused by the virus but also addresses the heart’s increased workload and structural complications, ultimately easing the burden of HF in patients with COVID-19.

## 3. Preventive measures for long-term COVID-induced HF

In order to prevent the adverse association between long-term COVID-19 and HF, the following preventive strategies may be beneficial:

### 3.1. Health education

Raising awareness through health education about the potential risk of HF following long-term COVID-19 infection is crucial. People need to understand the risks of HF after COVID-19 infection and take appropriate preventive and management measures. First, providing essential information about HF, including its causes, symptoms, diagnosis, prevention, and treatment, is essential.^[26]^ Explaining that HF is a pathological condition characterized by decreased cardiac function and can be caused by various factors, including COVID-19 infection. Simultaneously, educating individuals about the risk factors and causes of HF following COVID-19 infection is crucial. This may include the virus’s direct impact on the heart, inflammatory response, blood clot formation, and myocardial injury. Emphasizing the importance of maintaining a healthy lifestyle, including regular exercise, a balanced diet, sufficient sleep, and stress reduction, is vital. These measures can lower the risk of HF, promote early identification, and facilitate prompt treatment.

High-quality health education and awareness campaigns can enhance people’s understanding of long-term COVID-induced HF, encouraging them to take appropriate preventive measures and actively manage their health.^[32]^ This will help minimize the negative impact of long-term COVID-19 and HF, improving patients’ quality of life and prognosis. Increasing public awareness and risk perception of COVID-19 and promoting proper protective measures, such as wearing masks, frequent handwashing, and maintaining social distancing can help reduce the risk of infection. Following public health guidelines, actively participating in preventive measures, and getting vaccinated regularly are essential steps to protect against COVID-19.

### 3.2. Early screening and intervention

Early cardiovascular screening and assessment are crucial for patients with long-term COVID-19. This includes, but is not limited to, tests such as electrocardiograms and cardiac biomarkers.^[33]^ For confirmed COVID-19 patients, timely treatment and close medical monitoring can significantly reduce the risk of cardiac inflammation and myocardial injury.^[34]^ For high-risk individuals, such as those with preexisting HF or other cardiovascular disease risks, emphasis should be placed on basic preventive measures, such as wearing masks, frequent handwashing, and maintaining social distancing.

In both acute and chronic settings, cardiologic evaluations are imperative for managing patient care effectively. Acutely, any patient presenting with symptoms suggestive of cardiac involvement such as chest pain, dyspnea, or palpitations, especially in the context of recent COVID-19 infection, should undergo a comprehensive cardiovascular assessment. This includes the measurement of cardiac biomarkers like troponin, which is crucial for diagnosing myocardial injury. Chronic conditions that necessitate ongoing cardiological oversight include persistent or late-onset symptoms following COVID-19 recovery, such as continued chest discomfort, cardiac fatigue, and arrhythmias. For these patients, periodic evaluations using electrocardiograms, echocardiography, and serum biomarkers are recommended to monitor potential long-term cardiovascular sequelae. Additionally, patients with a history of cardiovascular disease or those presenting new risk factors during or after COVID-19 recovery should be closely monitored with regular cardiac assessments to preemptively manage and mitigate the risk of exacerbating preexisting conditions or developing new cardiac complications.

### 3.3. Vaccination

COVID-19 vaccination is a key measure to prevent COVID-19 infection and its related complications, including preventing the occurrence of cardiac inflammation and HF.^[35]^ Strict adherence to COVID-19 preventive measures, such as getting vaccinated, wearing masks, frequent handwashing, and maintaining social distancing, can reduce the risk of infection and, consequently, the likelihood of developing HF. Actively getting vaccinated against COVID-19 is an important step in reducing infection and the severity of the disease, especially for HF patients and high-risk individuals.

### 3.4. Preventing thrombosis

Preventing thrombosis is crucial for mitigating the spectrum of cardiovascular complications in long-term COVID-19, including HF. Thrombotic events significantly contribute to cardiovascular pathology; however, the prevention of such events encompasses more than just reducing the risk of HF. It is important to differentiate between arterial and venous thrombosis in this context. Arterial thrombosis, often leading to acute events like myocardial infarction and stroke, and venous thrombosis, which includes conditions such as deep vein thrombosis and pulmonary embolism, both require distinct preventive strategies. These strategies should be tailored to individual risk profiles and may include anticoagulation, physical activity, and lifestyle modifications, overseen by healthcare professionals to ensure optimal management of both arterial and venous thrombotic risks. For individuals with long-term COVID-19, active participation in moderate physical activities and exercise, such as walking, stretching exercises, or appropriate aerobic exercises, is recommended. Physical activity promotes blood circulation and reduces the risk of thrombosis. It is also advised to change positions timely as prolonged immobility may increase the risk of thrombosis. For long-term COVID-19 patients, especially those confined to bed rest, regular changes in body position, including body rotation and limb movements, are recommended to enhance blood circulation. For long-term COVID-19 patients, particularly those with high-risk factors such as severe illness, prolonged bed rest, or a history of venous thromboembolism, doctors may consider prescribing anticoagulant medications such as heparin or warfarin. These medications help prevent thrombosis.

It is crucial that these measures be guided and managed by healthcare professionals based on individual circumstances. Each patient’s needs and risk factors are unique, so it is advisable to communicate with doctors to develop a personalized plan for preventing thrombosis.^[25,36,37]^ Regular follow-up and assessments are also vital to ensuring the effectiveness of preventive measures. Adopting preventive measures such as adequate fluid intake, reasonable physical activity, and the use of anticoagulant medications can help lower the risk of thrombosis and reduce the occurrence of cardiovascular events. For patients who develop HF following a COVID-19 infection, cardiac rehabilitation can aid in improving heart function, managing the condition, and enhancing their quality of life.

Recent studies have demonstrated that the continuation of prophylactic anticoagulant therapy postdischarge can significantly reduce the risk of mortality within 30 days for patients recovering from COVID-19, without an increase in bleeding incidents, confirming its safety profile. Extended duration of prophylactic anticoagulation has been shown to further decrease the risk of venous thromboembolism among these patients. However, there is currently no consensus on guidelines advocating for extended prophylactic anticoagulation for all discharged COVID-19 patients.^[38]^ These findings suggest that postdischarge anticoagulant therapy could be a critical component of the management strategy for long-term COVID-19 patients, particularly those at elevated risk for thrombotic events. Healthcare professionals should consider individual risk assessments and patient-specific factors when deciding on the duration and type of anticoagulation therapy to optimize both safety and efficacy in preventing thrombotic complications.

### 3.5. Actively managing risk factors

Controlling common cardiovascular risk factors such as hypertension, diabetes, and hyperlipidemia, as well as adopting a healthy lifestyle, including a balanced diet and moderate exercise, is essential.^[39,40]^ Monitoring and follow-up of patients are crucial, and for those with existing HF, enhanced medical management is necessary, including regular follow-ups, periodic cardiac function assessments, and monitoring of disease progression. Additionally, managing other cardiovascular disease factors such as hypertension is important.

## 4. Reflection and value guidance

Concerning the issue of long-term COVID-induced HF, it is crucial to pay more attention to the long-term sequelae and complications of COVID-19 infection. In the past, COVID-19 was predominantly viewed as an acute disease, but now we realize that it can lead to long-term health problems, such as HF. This highlights the need for continuous monitoring, diagnosis, and treatment for patients with long-term COVID-19.^[39,40]^ Long-term COVID-19 patients often require multidisciplinary support and management, including internal medicine specialists, respiratory physicians, cardiologists, rehabilitation therapists, and mental health experts. This collaborative approach ensures that patients receive comprehensive medical and rehabilitation services, and personalized treatment plans are formulated. Early identification and appropriate interventions are crucial, including early detection of HF symptoms and signs and implementing appropriate treatment measures to alleviate symptoms, prevent progression, and improve prognosis.

A more comprehensive understanding of the complexity and severity of long-term COVID-induced HF and the adoption of appropriate preventive and management measures are essential to improve patients’ quality of life and prognosis and raise public awareness and concern about this issue.

## 5. Conclusions

As the COVID-19 pandemic persists, “long-term COVID-19” or PASC has emerged as a significant focus in global health. This review delves deeply into the long-term cardiovascular implications of COVID-19, particularly the risk of HF.

### 5.1. Cardiovascular implications

Research indicates that COVID-19, beyond its impact on the respiratory system, has significant effects on the cardiovascular system, leading to myocardial injuries, abnormal inflammatory responses, and thrombosis formation.

### 5.2. Preventive strategies

To mitigate the adverse association between prolonged COVID-19 infection and HF, health education and raising public awareness about the potential risks of HF are paramount.

### 5.3. Future research

While we have gained insights into the long-term effects of COVID-19, further studies are essential to determine the best preventive and therapeutic strategies to reduce the risk of cardiovascular complications.

We acknowledge the limitations of our study. Initially, challenges arose from inaccessible articles and language barriers, hindering our access to all pertinent publications. Furthermore, there was notable variability in the quality of the included studies, with some offering limited data. This diversity in methodology and data completeness may have impacted the reliability of our analysis and the applicability of our conclusions.

In conclusion, as the widespread vaccination against COVID-19 and therapeutic strategies continue to evolve, the prevention and management of cardiovascular complications remain a pivotal challenge in the medical and health sector. Collaboration among healthcare professionals, researchers, and the public is crucial to ensure optimal cardiovascular health outcomes.

## Author contributions

**Conceptualization:** Gang Li, Zhangqing Ren.

**Supervision:** Gang Li.

**Writing—original draft:** Gang Li.

**Writing—review & editing:** Zhangqing Ren.
